# Improved child marriage laws and its association with changing attitudes and experiences of intimate partner violence: a comparative multi-national study

**DOI:** 10.7189/jogh.10.010707

**Published:** 2020-06

**Authors:** Negar Omidakhsh, Jody Heymann

**Affiliations:** WORLD Policy Analysis Center, University of California, Los Angeles, Los Angeles, California, USA

## Abstract

**Background:**

Intimate partner violence (IPV) is a multi-national problem with many health consequences. Some research suggests that reducing rates of child marriage can improve gender norms and health outcomes related to IPV. Here, we examine whether changes in national child marriage laws can improve attitudes about domestic violence and reduce intimate partner violence at scale.

**Methods:**

Data on attitudes towards violence and violence experienced were obtained from the Demographic and Health Surveys (DHS) and longitudinal data on child marriage policy from WORLD and MACHEquity databases (1995-2012). Treatment countries were included if they improved their national child marriage policies from harmful (under 18) to more protective and control countries were included if they had a constant child-marriage policy that allowed girls to marry under the age of 18. Our final data set included 5 treatment and 14 control countries for women’s outcomes, 2 treatment and 9 control countries for men’s outcomes and 2 treatment and 7 control countries for IPV outcomes (for which fewer countries collect data). We combined individual level responses to five questions on attitudes about domestic violence to create a scale from 0 (always unacceptable) to 5 (always acceptable). All analyses employed a difference-in-differences approach adjusting for individual and country level predictors.

**Results:**

Data were available for 532 255 women, of which 96 414 also completed the domestic violence modules, and 104 704 men. National changes to a protective child marriage policy were associated with improved attitudes towards violence among women (-0.21 points, 95% confidence interval (CI) = -0.28, -0.14) and men (-0.98 points, 95% CI = -1.13, -0.83). Additionally, the risk of women experiencing physical and sexual abuse reduced by a greater proportion in treatment compared to control countries (odds ratio OR = 0.65, 95% CI = 0.50, 0.84; OR = 0.63, 95% CI = 0.45, 0.88, respectively).

**Conclusions:**

Our large multi-national study is the first of its kind to critically evaluate the role of national policy on attitudes towards and experiences of IPV among both men and women, and finds that these laws have protective outcomes. Our results are consistent with the hypothesis that gender egalitarian laws positively influence norms and health at the national level.

The WHO estimates that approximately 1 in 3 women worldwide have experienced physical or sexual IPV in their lifetime and that up to 58% of women have experienced some form of emotionally abusive behavior in the past year[1]. Violence against women has substantial negative impacts on their health, their economic status and progress towards gender equality [2,3]. Adolescent girls are especially vulnerable to IPV [4-7]. However, though much research over the last two decades has explored the impact of and risk factors associated with IPV, there is little empirical evidence on whether programmatic efforts or country-level policy changes are effective at reducing IPV [8]. Studies suggest that child marriage, defined as the marriage of girls under 18, is a strong risk factor for IPV [7,9]. Moreover, several studies have found that women who marry over the age of 18 are more likely to know about and participate in the planning of their marriage, reject wife beatings and are at lower risk of IPV [4,10]. This raises the question of whether national child marriage laws, either in and of themselves, or as catalysts for broad dialogue on gender-equitable norms, can reduce IPV at scale or improve IPV-related norms.

Child marriage is a multi-national problem that cuts across cultures and religions and has become widely regarded as a harmful traditional practice and human rights violation [11,12]. The global burden is substantial and each year over 15 million girls are married before their 18^th^ birthday [5]. Child marriage is associated with adverse reproductive, child and mental health outcomes aside from IPV [1,13-17]. Girls who marry young are more likely to have high risk adolescent pregnancies and, in consequence, their children are 60% more likely to die before their first birthday than the children of women who marry after age 18 [13-15]. Another study found that women who were married as children had higher rates of lifetime psychiatric disorders and were almost twice as likely to have both nicotine and drug dependence [17]. Child marriage may contribute to IPV through several potential mechanisms. The first is that child marriage often represents an inherent lack of autonomy and power imbalance within relationships, each of which have been independently found to be a risk factor for IPV [18]. Child marriages are often negotiated by parents who raise their children in households that undervalue women and, consequently, these young girls may harbor harmful beliefs regarding the justification of violence towards women, increasing their risk of experiencing IPV [4,19]. Finally, these marriages often result in large spousal age- and education-gaps, which can further fuel the power imbalance within a relationship and lead to greater marital violence [8,20].

Egalitarian gender-based policies and laws have previously been found to positively impact norms regarding women’s equality and empowerment at the national and sub-national level [21-23]. There is evidence of an association between violence norms and adverse health outcomes, including IPV, and some studies have suggested that one cannot fully understand the causes of IPV without first examining the social context in which it occurs [24-26]. Interventions aimed at addressing and transforming cultural norms that support violent behavior can help reduce and prevent IPV [27].

Enforcing laws that prohibit child marriage can both reduce high risk child marriages and discredit unequal and hazardous gender expectations. In addition, child marriage laws are often followed by national campaigns that aim to change social norms and create a protective environment for girls and their communities [28]. These initiatives often include targeted mass media campaigns and community-wide education and outreach interventions, which have a positive effect on gender norms and attitudes towards violence against women [29-33]. Past literature has observed a positive impact of community-based interventions on IPV and IPV normative outcomes among both women and men [8,29,32-34].

This study examines the relationship between national child marriage laws, attitudes about domestic violence and intimate partner violence. We hypothesize that legislative changes to a more protective child marriage policy will reduce the acceptability of using violence against women among women and men and reduce the odds of women having experienced intimate partner violence within the last year. For the present study, we utilize a difference-in-differences approach to estimate the association between changes in child marriage law, attitudes towards and experiences of intimate partner violence among women and men in 19 low and middle income countries.

## METHODS

### Data sources

Individual-level outcome data on attitudes and intimate partner violence were obtained from the Demographic and Health Surveys (DHS). The DHS are nationally representative, cross-sectional, household survey data from low- and middle-income countries and are obtained through a stratified two-stage cluster design [35]. In the first stage, communities within defined regions are randomly selected from census files and, in the second stage, households are randomly sampled from household rosters within these communities. Selected households are approached for in-person interviews and data are collected for all female residents between the ages of 15-49, men ranging from 15-49, 15-54, 15-59, or 15-64 years old depending on the country, and children under 5 years. In countries where a domestic violence module is administered, one eligible woman (aged 15-49) is selected from approximately 50% of all eligible households. More details on the DHS sampling strategy and comparability across surveys and waves can be found elsewhere [35].

For the present study, we utilized questions from the DHS women’s and men’s questionnaires from 19 low and middle income countries. To be included as a treatment country, countries had to have experienced a change in national child marriage policy from harmful (under 18) to more protective (18 with no possibility of exceptions based on parental consent, with the exception of Mozambique whose legal age of marriage changed to 16, herein referred to as treatment countries). To be included as a control country, countries had to have had a constant child-marriage policy that allowed girls to marry under the age of 18 either generally (without regard for special circumstances) or with parental consent (herein referred to as control countries). Both treatment and control countries had to have at least two available DHS surveys administered between 2000-2016. Our final sample included 5 treatment and 14 control countries (**Table 1**).

**Table 1:** Country and child marriage policy descriptions for treatment and control countries, Demographic and Health Surveys

Treated countries	DHS survey years	Number of women	Number of men	Women included in DV module	Prior legal age of marriage	Year of law change	Current legal age of marriage	Before and after legal age of child marriage
Benin	2001, 2011/12	22 818	6869	-	15	2004	18	Changed from 15 to 18 y old
DRC	2007, 2013/14	28 822	-	7647	15	2009	18	Changed from 15 to 18 y old
Egypt	2005, 2014	41 236	-	11 543	16	2008	18	Changed from 16 to 18 y old
Guinea	2005, 2012	17 096	-	-	17	2008	18	Changed from 17 to 18 y old
Mozambique	2003/4, 2011	26 163	6018	-	14	2004	16	Changed from 14 to 16 y old
**Control countries**					**Legal age of marriage (unchanged)**			
Armenia	2005, 2015/16	12 682	4202	-	17	-	-	No change
Burkina Faso	2003, 2010	29 564	9689	-	17	-	-	No change
Cameroon	2004, 2011	26 082	-	5966	15	-	-	No change
Haiti	2005/6, 2012	25 044	-	8214	15	-	-	No change
Honduras	2005/6, 2011/12	42 705	-	22 128	16	-	-	No change
Indonesia	2002/3, 2012	75 090	15 427	-	16	-	-	No change
Kenya	2003, 2008/9	16 639	6604	8237	16	-	-	No change
Lesotho	2004/5, 2014	13 716	5121	-	16	-	-	No change
Malawi	2004/5, 2015	36 260	10 223	12 091	15	-	-	No change
Niger	2006, 2012	20 383	-	-	15	-	-	No change
Senegal	2005, 2014/15	31 941	-	-	16	-	-	No change
Tanzania	2004/5, 2015/16	23 595	6149	-	15	-	-	No change
Zambia	2007, 2013/14	23 557	19 535	11 589	16	-	-	No change
Zimbabwe	2005/6, 2015	18 862	14 867	8999	16	-	-	No change
**Total**		**532** **255**	**104** **704**	**96** **414**				

DRC – Democratic Republic of the Congo

Longitudinal data on child marriage policy was extracted from WORLD and MACHEquity databases. These data sources contain national marriage laws for all 193 United Nations (UN) member states from the period 1995-2012, and code policy based on a collection of primary-source legislation, United Nations reports and country specific reports sent to international bodies and monitoring committees. A deeper discussion of methodology is reported elsewhere [36]. For countries with DHS surveys from 2013-2016, we independently searched for legislation to determine if their child marriage policies had changed since 2012. For each country with an established minimum age of marriage, data was coded on the exact age for which marriage was permitted generally, with parental consent exceptions and with customary or religious law exceptions.

Our final data set was created by linking individual-level DHS data to country-level policy across all countries and time periods and contained 532 255 female and 104 704 male observations. Among all women, 96 414 were interviewed for the domestic violence module from 2 treatment and 7 control countries. To account for country-specific survey sample designs, and per DHS guidelines on using pooled data, standard weights for each respondent were de-normalized and then re-weighted using UN population division measures on total females and men aged 15-49 in the country at the time of the survey [37,38].

### Measures

Our exposure of interest was the legal age of marriage for girls for each country and year. For our outcome variable, we utilized questions on attitudes about domestic violence, which were asked of all women and men. Women and men were asked the following question, during face-to-face interviews, “In your opinion, is a husband justified in hitting or beating his wife in the following situations?”: “if she goes out without telling him”, “if she neglects the children”, “if she argues with him”, “if she refuses to have sex with him” and “if she burns the food.” Possible responses included yes (coded here as 1), no (coded here as 0) or I don’t know for each question. No more than 3% of respondents in each survey answered “*I don’t know*” to each item; these were grouped with those answering No. All violence justification questions were summed to create a scale from 0 to 5, with 0 indicating the respondent felt violence was never justified and 5 indicating that it was justified in all scenarios. Thus, a one unit decrease on our scale would imply that a person who previously felt it was justified to hit or beat a wife in one of the abovementioned scenarios, no longer believes it is justified to do so.

Not all DHS surveys include the domestic violence module; in the present study only 9 countries administered the domestic violence module in both time periods of interest. Within these countries, women currently in union were asked if they experienced emotional, physical, or sexual violence. With respect to emotional violence respondents were asked: has your spouse ever (1) humiliated you, (2) threatened you with harm, (3) insulted you or made you feel bad. With respect to physical violence respondents were asked: has your spouse ever (1) pushed, shook or threw something at you, (2) slapped you, (3) punched you with their fist or something harmful, (4) kicked or dragged you, (5) tried to strangle or burn you, (6) threatened you with a knife or other weapon, (7) attacked you with a knife or weapon, (8) twisted your arm or pulled your hair; for sexual violence: has your spouse ever (1) physically forced sex when not wanted, (2) forced other sexual acts when not wanted. Possible responses to each of the questions above varied by country. For the earliest phases of Egypt, Cameroon, Kenya and Malawi, respondents could answer “yes” or “no” and were then asked a subsequent question on the frequency of attacks during the last 12 months. If women had at least one attack in the last 12 months, they were coded as 1 for either emotional abuse, physical abuse, or sexual abuse, otherwise they were coded as 0. For all other countries, responses to each question were recorded as either “never” (coded as 0), “often” (coded as 1), “sometimes” (coded as 1), “yes, but not in the last 12 months” (coded as 0) or “yes, but frequency in the last 12 months missing” (coded as missing). Responses to questions on emotional violence were not available for Honduras during the first time period and thus Honduras was excluded from all analyses with emotional violence outcomes.

The inclusion of covariates was based on a review of the literature of child marriage, norms and IPV [7,19,20,39-42]. In our adjusted analyses, we included respondent’s age (grouped in 5-year increments), respondent’s place of residence (defined as urban or rural), respondent’s educational attainment (no education, incomplete primary, complete primary, incomplete secondary, complete secondary, higher), respondent’s current working status (yes or no), respondent’s marital status (never married, married, living with partner, previously married), respondent’s birth year, log GDP per capita, female unemployment rate at the time of survey administration (only included in our women’s attitudes analyses), respondent and partner age difference (only included in our IPV analyses) and the DHS wealth index quintile, a measure of household socioeconomic status (SES), calculated using information regarding household assets (eg, television or radio), housing construction, water access and sanitation facilities[43]. Other covariates that were considered but not included in our model, as they did not change point estimates by greater than 10%, were parity, age at first intercourse and access to contraception (defined as women’s met need for contraception).

### Analyses

All analyses employed a difference in differences approach to estimate the effect that changing child marriage policies have on attitudes towards violence and emotional, physical or sexual IPV experienced within the last 12 months. This quasi-experimental analytical approach allows one to study causal relationships in settings where randomized control trials are not possible and requires data from pre- and post-intervention, thus allowing us to make use of longitudinal data to estimate casual effects. By comparing the changes in outcomes over time between treatment groups (those that undergo a specific intervention) and control groups (those that do not) we were able to determine the effects that laws against child marriage have by measuring the changes in our outcomes occurring in countries that improved their child marriage laws (treatment countries) relative to changes in our outcomes occurring in countries that continued to legally permit child marriage throughout the study period (control countries). All models included fixed effects for country and survey year in an attempt to adjust for unobserved time-invariant confounders specific to each country as well as temporal normative trends that are shared across countries. All analyses on IPV outcomes were restricted to women currently married or living with their partner.

Our first model examines the association between changes in marriage policy and our outcomes using sample weights and fixed effects for country and survey year only (Model 1; univariate). In our second model, we included all covariates (Model 2; multivariate). We incorporated robust standard errors in all of our models to account for clustering at the country level.

In addition to the above analyses, we performed sensitivity analyses that restricted Mozambique from our treatment countries as their minimum age of marriage changed from 14 to 16, as opposed to the 18+ age restriction set in our other treatment countries. Girls younger than 18 years of age may not be able to give their free and full consent to marriage; therefore, the same positive normative changes may not be observed in Mozambique relative to the other treatment countries that increased the minimum age of marriage to 18. All analyses were run using Stata Statistical Software: Release 14, College Station TX, USA. All data used for the present study is de-identified and publicly accessible online; thus it did not require review from an internal ethics committee.

## RESULTS

**Table 1** provides an overview of all treatment and control countries included in the present study. All but one treatment country experienced a protective change in child marriage policy from under 18 to 18 years of age without parental exception loopholes. Only a subset of these countries also collected data from men (2 treatment and 9 control) and/or administered the domestic violence module (2 treatment and 7 control) in both time periods.

**Table 2** and **Table 3** compares the change in the attitudes towards violence, measured through our violence scale, in treatment and control countries among women and men, respectively. In **Table 2**, our multivariate model (model 2) revealed that women from treatment countries, on average, experienced a decrease of 0.21 points [95% confidence interval (CI): -0.28, -0.14] in our attitudes towards violence scale (where 0 indicates wife beatings are never justified and 5 indicates they are justified in all scenarios). In **Table 3**, in our fully adjusted model, men from treatment countries had a mean change on the attitude scale of -0.98 points (95% CI = -1.13, -0.83) greater than the mean change in control countries. In every age group, we found that changes in child marriage policy had a significant positive effect on IPV-related attitudes among men and that very young men (ages 15 to 19) in treatment countries experienced the largest mean change (1.89 point decrease, 95% CI = -2.48, -1.29).

**Table 2:** Association between change in legal age of marriage and changes in violence attitudes scale, WOMEN aged 15-49 Demographic and Health Surveys

	Univariate* (N = 531 803)	Multivariate† (N = 524 925)
**Exposure:**		
Change in policy to 18+ (DID estimator)	-0.20 (-0.28, -0.13)‡	-0.21 (-0.28, -0.14)‡
**Individual, household and country level predictors:**		
Respondent age: 20-24 years		0.01 (-0.01, 0.04)
Respondent age: 25-29 years		0.01 (-0.03, 0.05)
Respondent age: 30-34 years		0.04 (-0.02, 0.09)
Respondent age: 35-39 years		0.08 (0.01, 0.15)§
Respondent age: 40-44 years		0.16 (0.07, 0.25)§
Respondent age: 45-49 years		0.22 (0.11, 0.32)§
Rural residency status		0.13 (0.11, 0.15)‡
Educational attainment: incomplete primary		-0.16 (-0.18, -0.14)‡
Educational attainment: complete primary		-0.28 (-0.30, -0.25)‡
Educational attainment: incomplete secondary		-0.38 (-0.41, -0.36)‡
Educational attainment: complete secondary		-0.57 (-0.59, -0.54)‡
Educational attainment: higher		-0.73 (-0.76, -0.70)‡
Household SES 2^nd^ quintile		-0.09 (-0.11, -0.06)‡
Household SES 3^rd^ quintile		-0.17 (-0.19, -0.14)‡
Household SES 4^th^ quintile		-0.27 (-0.30, -0.25)‡
Household SES 5^th^ quintile		-0.42 (-0.45, -0.39)‡
Respondent's currently working		0.03 (0.01, 0.04)‡
Respondent's marital status: married		0.15 (0.13, 0.17)‡
Respondent's marital status: living with a partner		0.11 (0.08, 0.13)‡
Respondent's marital status: previously married or living with a partner		0.08 (0.06, 0.11)‡
Respondent year of birth		0.01 (0.01, 0.02)‡
Log GDP per capita		0.28 (-0.06, 0.63)
Female unemployment rate		-0.01 (-0.01, 0.01)

GDP – gross domestic product

*Univariate model includes country and year fixed effects.

†Multivariate model additionally controlled for measured individual, household and country-level characteristics.

‡*P* < 0.001.

§*P* < 0.05.

**Table 3:** Association between change in legal age of marriage and changes in violence attitudes scale, MEN aged 15-49 Demographic and Health Surveys

	Univariate* (N = 104 604)	Multivariate† (N = 102 034)
**Exposure:**		
Change in policy to 18+ (DID estimator)	-.71 (-0.86, -0.56)‡	-0.98 (-1.13, -0.83)‡
**Individual, household and country level predictors:**		
Respondent age: 20-24 years		-0.10 (-0.14, -0.06)‡
Respondent age: 25-29 years		-0.12 (-0.19, -0.06)‡
Respondent age: 30-34 years		-0.11 (-0.20, -0.01)§
Respondent age: 35-39 years		-0.06 (-0.18, 0.06)
Respondent age: 40-44 years		0.02 (-0.13, 0.17)
Respondent age: 45-49 years		0.03 (-0.15, 0.21)
Rural residency status		0.06 (0.03, 0.09)‡
Educational attainment: incomplete primary		-0.04 (-0.08, 0.00)
Educational attainment: complete primary		-0.13 (-0.18, -0.09)‡
Educational attainment: incomplete secondary		-0.26 (-0.30, -0.22)‡
Educational attainment: complete secondary		-0.36 (-0.41, -0.31)‡
Educational attainment: higher		-0.49 (-0.54, -0.44)‡
Household SES 2^nd^ quintile		-0.06 (-0.10, -0.03)‡
Household SES 3^rd^ quintile		-0.07 (-0.11, -0.03)‡
Household SES 4^th^ quintile		-0.16 (-0.19, -0.12)‡
Household SES 5^th^ quintile		-0.26 (-0.30, -0.22)‡
Respondent's currently working		0.09 (0.07, 0.12)‡
Respondent's marital status: married		-0.04 (-0.07, -0.01)§
Respondent's marital status: living with a partner		-0.04 (-0.09, 0.01)
Respondent's marital status: previously married or living with a partner		0.15 (0.09, 0.20)‡
Respondent year of birth		0.01 (0.01, 0.02)‡
Log GDP per capita		0.65 (0.26, 1.03)§

SES – socio-economic status

*Univariate model includes country and year fixed effects.

†Multivariate model additionally controlled for measured individual, household and country-level characteristics.

‡*P* < 0.001.

§*P* < 0.05.

**Table 4** displays the results of our IPV analyses and gives the average change in odds ratios (OR) of women from treatment countries experiencing either emotional, physical or sexual IPV compared to women in control countries. Women in treatment countries have a reduced risk of experiencing physical violence (OR = 0.65, 95% CI = 0.50, 0.84) and sexual violence in the last 12 months (OR = 0.63, 95% CI = 0.45, 0.88). Odds of experiencing emotional violence increased among women in treatment countries compared to control countries after all adjustments (OR = 1.54, 95% CI = 1.12, 2.11).

**Table 4:** Association between change in legal age of marriage and changes in odds of experiencing various types of violence in the last 12 months, WOMEN aged 15-49 Demographic and Health Surveys

	Emotional violence*		Physical violence		Sexual violence	
	**Univariate**† **(N = 74 138)**	**Multivariate**‡ **(N = 73 637)**	**Univariate**† **(N = 96 224)**	**Multivariate**‡ **(N = 95 698)**	**Univariate**† **(N = 96 248)**	**Multivariate**‡ **(N = 95 754)**
Change in policy to 18+ (DID estimator)	0.60 (0.47, 0.77)§	1.54 (1.12, 2.11)‖	0.59 (0.47, 0.74)§	0.65 (0.50, 0.84)‖	0.46 (0.34, 0.62)§	0.63 (0.45, 0.88)‖
**Individual, household and country level predictors:**						
Respondent age: 20-24 years		1.15 (1.01, 1.31)‖		1.24 (1.10, 1.39)§		1.08 (0.94, 1.26)
Respondent age: 25-29 years		1.13 (0.94, 1.37)		1.23 (1.04, 1.46)‖		1.15 (0.94, 1.42)
Respondent age: 30-34 years		1.03 (0.79, 1.34)		1.23 (0.97, 1.55)		1.22 (0.90, 1.64)
Respondent age: 35-39 years		0.88 (0.62, 1.24)		1.13 (0.84, 1.54)		1.22 (0.83, 1.80)
Respondent age: 40-44 years		0.80 (0.52, 1.23)		1.23 (0.85, 1.79)		1.20 (0.75, 1.94)
Respondent age: 45-49 years		0.67 (0.40, 1.12)		1.12 (0.71, 1.77)		1.13 (0.63, 2.02)
Rural residency status		0.79 (0.73, 0.86)§		0.77 (0.71, 0.82)§		0.89 (0.80, 0.98)‖
Educational attainment: incomplete primary		1.15 (1.06, 1.24)§		1.20 (1.12, 1.29)§		1.37 (1.24, 1.50)§
Educational attainment: complete primary		1.01 (0.91, 1.11)		1.02 (0.94, 1.11)		1.22 (1.09, 1.37)‖
Educational attainment: incomplete secondary		1.01 (0.92, 1.11)		1.09 (1.01, 1.19)‖		1.18 (1.05, 1.32)‖
Educational attainment: complete secondary		0.66 (0.58, 0.74)§		0.68 (0.60, 0.76)§		0.71 (0.59, 0.85)§
Educational attainment: higher		0.54 (0.45, 0.64)§		0.51 (0.43, 0.61)§		0.58 (0.46, 0.73)§
Household SES 2^nd^ quintile		0.96 (0.89, 1.03)		0.99 (0.92, 1.05)		0.98 (0.90, 1.06)
Household SES 3^rd^ quintile		0.97 (0.90, 1.05)		1.01 (0.94, 1.09)		0.99 (0.91, 1.09)
Household SES 4^th^ quintile		0.88 (0.80, 0.97)‖		0.91 (0.83, 0.99)‖		0.87 (0.78, 0.98)‖
Household SES 5^th^ quintile		0.70 (0.63, 0.79)§		0.74 (0.66, 0.82)§		0.67 (0.58, 0.78)§
Respondent's currently working		1.26 (1.19, 1.34)§		1.23 (1.17, 1.29)§		1.41 (1.32, 1.51)§
Marital age gap: 1-5 years		0.95 (0.86, 1.05)		0.91 (0.85, 0.99)‖		1.04 (0.93, 1.16)
Marital age gap: 6-10 years		0.96 (0.87, 1.06)		0.88 (0.82, 0.96)‖		0.98 (0.88, 1.11)
Marital age gap: 11-15 years		1.03 (0.92, 1.16)		0.80 (0.72, 0.88)§		0.99 (0.86, 1.14)
Marital age gap: 16-20 years		1.08 (0.93, 1.25)		0.83 (0.72, 0.94)‖		0.86 (0.72, 1.03)
Marital age gap: 21+ years		1.04 (0.89, 1.22)		0.72 (0.62, 0.83)§		0.96 (0.79, 1.16)
Respondent year of birth		0.99 (0.97, 1.00)		1.02 (1.01, 1.04)‖		1.01 (0.99, 1.03)
Log GDP per capita		86 732.96 (77 62.44, 969 103.70)§		2.26 (0.44, 11.60)		37.5 (5.16, 273.03)§

SES – socio-economic status, GDP – gross domestic product

*Honduras excluded from control countries as questions regarding emotional violence were not asked.

†Univariate model includes country and year fixed effects.

‡Multivariate model additionally controlled for measured individual, household and country-level characteristics.

§*P* < 0.001.

‖*P* < 0.05.

Results of the sensitivity analyses that excluded Mozambique from our treatment countries revealed only marginal differences in outcomes (less than 10%). However, given reduced sample sizes, confidence intervals widened in our men’s sample.

## DISCUSSION

Our longitudinal multi-national study found that national changes to limit child marriage were associated with improved norms against violence among both men and women and were protective against both sexual and physical violence in the last year. While changes to a protective child marriage policy increase the odds of women reporting emotional violence, it is unclear whether this is due to an increased awareness leading to increased reporting or an actual increase in emotional violence in the presence of declining physical and sexual violence. To our knowledge, this is the first study to examine the association between passage of child marriage laws and normative and IPV-related health outcomes at scale, and our results are consistent with the hypothesis that gender egalitarian laws positively impact norms and health at the national level.

Laws can have impact both through their direct enforcement and through normative change. The evidence on enforcement of child marriage laws is limited [44]; however, analysis of our data found that the rate of child marriage reduced by an average of 6% in treatment countries. Reducing rates of child marriage itself may result in a lower risk of IPV; indeed, a recent study found that young girls who married as children were more likely to have experienced physical and/or sexual IPV in the last year compared to young women who married as adults [7].

Child marriage laws, combined with advocacy efforts surrounding their enactment, may also prevent violence by improving gender-equitable attitudes towards IPV. The combination of national legislation and widespread community outreach may have fostered the positive changes observed in our study. The countries with a decrease on our violence attitudes scale, Egypt, Benin, and Mozambique, had strong community outreach and mobilization efforts surrounding law enactment.

After Egypt’s family law criminalized the marriage of girls under 18, a five-year national strategy led by the National Population Council to end child marriage was launched [45]. The goal of the program is to reduce the prevalence of child marriage by 50% over five years through two main approaches. The first is a rights-based approach which ensures that constitutional laws that ban early marriage are also upheld at the religious and customary level. The second is a partnership approach designed to bring together government, public sector and civil society organizations to achieve 5 goals: empowering girls; supporting girls already married to minimize harmful impacts; continuing to update existing protective legislation to ensure it is working; educating and preparing girls to tackle family pressures to marry early and working with families and communities to educate them on the harmful consequences of child marriage [45].

Soon after the law change in Benin, the US Agency for International Development’s Women’s Legal Rights Initiative, along with other implementing partners, conducted an intensive grassroots awareness campaign reaching more than 100 000 people though educational brochures, films and radio broadcasts [46]. The country’s public awareness campaign disseminated booklets and brochures in local languages; produced and screened films on family law issues pertaining to child marriage; organized meetings with community leaders; trained hundreds of justice sector workers and paralegals on the new family law code preventing child marriage; held public forums and broadcasted political roundtables on women’s legal rights on television and radio [46].

Mozambique changed their child marriage laws in 2004, immediately after which the country established support services to victims of violence within police stations and health care facilities. A few years later, they mandated a police training module in all police academies on responding to violence against women and created the Zero Tolerance against Violence against Children Initiative.

All of these programs were created in the wake of each country’s new family law policies and harness the power of government and non-profit sectors to increase community awareness, girls’ and women’s opportunities and decision making power. In contrast, where implementation was ineffective and substantial civil society efforts absent, the same impact was not observed. The DRC was also the only treatment country in which the average rate of child marriage increased from time period 1 and 2 and had mean scores that increased over time.

Though we observed reduced point estimates for physical and sexual violence among all married women in our study, estimates on the youngest married women in our sample (ages 15-19) were not statistically significant. This may be because the policy helped delay marriage, and was effective at preventing IPV among girls who delayed marriage but not for the subsample who continued to marry early. This is consistent with other research including a recent study from Cote D’Ivoire which found community based gender dialogue groups and IPV prevention programming were ineffective at decreasing physical or sexual violence experienced among women married as child brides, despite these interventions having positive effects on non-child brides [47].

In contrast, women’s reports of emotional violence in the last year increased by 50% in treatment countries when compared to relative changes in control countries, after adjustment. Given the strong awareness and educational campaigns on the ground at the time of law change in treatment countries, women may now be more informed about emotional violence and, consequently, more likely to report it. Nonetheless, the observed increase in emotional violence is disturbing and raises important questions that these data can’t answer. It is possible that as women’s empowerment improves, emotional violence may increase. Some studies have found that the association between women’s autonomy and violence outcomes depends on the existing cultural context of patriarchy [48,49]. Future, prospective, studies are needed to properly identify the relationship between emotional violence and improved child marriage laws.

One strength of this study is the use of difference in difference methodology, which allows us to report on the average experience across a set of countries. The benefit of this approach is that it does not assume perfect implementation of the law at the country level, nor does it require perfect effectiveness. Rather, it allows us to examine whether a change in child marriage policy could work to improve norms and experiences around IPV in a country with average effectiveness and, if so, by how much; mimicking what most often occurs in the real world. Alternatively, the limitation is that though our analysis examines the effect from all countries combined, there is variability in the enforcement of these laws and some countries do better than others. There are a number of other important limitations to the present study. Our covariates were selected based on a review of the literature of child marriage and intimate partner violence; though we attempted to adjust for all relevant covariates, as with all non-experimental studies, there is still the possibility of confounding by unmeasured variables. These variables would need to be independently associated with child marriage policy change and our IPV and normative outcomes, which suggests that the pool of unaccounted potential confounders is limited. We rely on men and women’s accurate responses to questions on the justification of marital violence, and it is possible that respondents reported what they felt to be socially acceptable rather than their true beliefs. Not all treatment and control countries had DHS surveys available for the men’s or domestic violence questionnaires in both time periods, therefore different sets of countries were used for each analyses. Despite these limitations, our large multi-national study is the first of its kind to critically evaluate the role of national policy on IPV and attitudes towards domestic violence among both men and women, and finds that these laws have protective outcomes.

Laws prohibiting child marriage are often one component of a revised family law package which also include improvements to divorce and separation legislation. These laws often make it easier for women to exit harmful relationships; however, few studies have critically examined whether divorce laws, or divorce itself, works to prevent and protect women from IPV [50]. The nature of the law and norms may matter. Divorced women are often stigmatized, forced to relinquish their financial and custodial rights and unlikely to remarry, thus presenting an unfavorable alternative for many women in violent marriages. Further research should examine the impact of other policies likely to affect women’s equality in and out of marriage and partnerships, as well as women’s ability to autonomously choose to enter and exit unions.
